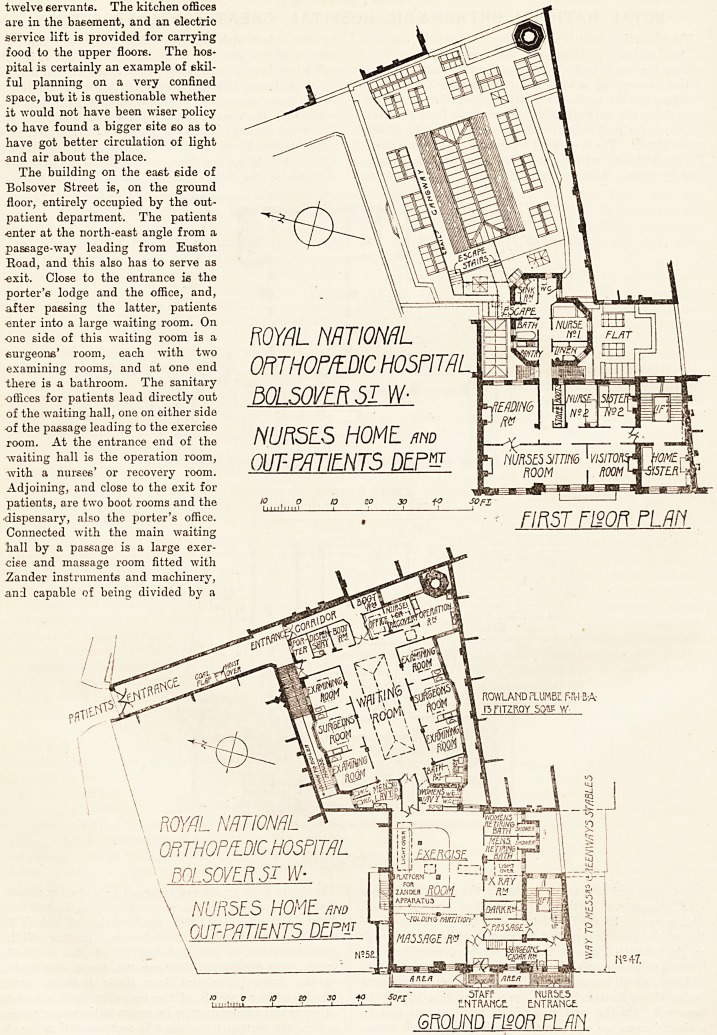# Royal National Orthopædic Hospital, Great Portland Street

**Published:** 1910-01-01

**Authors:** 


					January 1, 1910. THE HOSPITAL. 399
ROYAL NATIONAL ORTHOPAEDIC HOSPITAL, GREAT PORTLAND STREET,
This building, which has recently been rebuilt, consists
of two distinct portions?one the hospital proper, con-
taining the wards and a certain amount of staff accommo-
dation, occupying a eite running through from G,reat
Portland Street to Bolsover Street, and the other part
comprising the out-patient department and further accom-
modation for staff being on the other or east side of Bol-
sover Street. The hospital proper is entered from Great
Portland Street, but the ground floor on that side is occu-
pied by shops. The remainder of the ground floor contains
board room and offices, the rooms for the resident medical
staff, the matron's sitting room and office, and the nurses'
dining room. On the first floor and on the two floors above
the whole space is occupied by wards, which are, as well
as the restricted site will allow, arranged pavilion-wise.
There are three wards of varying sizes, a large ward for 24
children, which has its long axis east and west and one end
facing Great Portland Street, on which tide a balcony is
arranged. On the west side of the ward on one side is
a sisters' room, on the other side a bathroom, sink room,
and w.c. The position of these adjuncts must interfere
to some extent with the cross ventilation of the ward, but
in view of the very restricted nature of the site it is diffi-
cult to see how this could have been avoided. On the east
frontage, facing Bolsover Street, are two wards for 12 and
18 children respectively, with a sisters' room and two
sculleries. Each ward is provided with a bathroom and
the usual sanitary offices, and there is a lift, which is
available for all wards. The two ward blocks are detached
by an open lobby one from the other. The cubic space
allowed for the children in the wards is about 650 feet.
The upper floor wards are arranged in a similar mannerr
though the number in the wards is lees, each adult being
given 1,000 cubic feet. The total number of beds is 200'
in the wards and 13 on the balconies. In this building
accommodation is provided for 22 of the staff alto-
gether. On the fourth floor are two wards arranged as
on the floors below and four single isolation wards.
The fifth floor consists of the operation theatre and its
adjuncts and a laboratory and museum in the western wing,
and in the eastern wing there is accommodation for the
matron, assistant matron, night superintendent, cook, and
fflYfumiomb
omormwmt;
? ODD OcKS|f spD ? 0 Q J
CHILDREN CZ-H__ JF0 L-JS CMILDKLN5 r?I
j] u> n qj  1
mmouTu) czt <, 4-H?m- - -A WflfiD(Ncnm) 1?f
xnU ? d ? a n nil
31
FIRST FLOOFi PL/7N
N?53
B0L50VLR 5TRE.E.T ^ ROWLAND PLUMBfLTR IBA
CMINDFim PLAN- 13 FITZnor 5q"
400 THE HOSPITAL. January 1, 1910.
twelve servants. The kitchen offices
are in the basement, and an electric
service lift is provided for carrying
food to the upper floors. The hos-
pital is certainly an example of skil-
ful planning on a very confined
space, but it is questionable whether
it would not have been wiser policy
to have found a bigger site so as to
have got better circulation of light
and air about the place.
The building on the ea6t side of
Bolsover Street is, on the ground
floor, entirely occupied by the out-
patient department. The patients
?enter at the north-east angle from a
passage-way leading from Euston
Road, and this also has to serve as
?exit. Close to the entrance is the
porter's lodge and the office, and,
after passing the latter, patients
?enter into a large waiting room. On
one side of this waiting room is a
surgeons' room, each with two
examining rooms, and at one end
there is a bathroom. The sanitary
offices for patients lead directly out
of the waiting hall, one on either side
?of the passage leading to the exercise
room. At the entrance end of the
waiting hall is the operation room,
with a nurses' or recovery room.
Adjoining, and close to the exit for
patients, are two boot rooms and the
dispensary, also the porter's office.
Connected with the main waiting
hall by a passage is a large exer-
cise and massage room fitted with
Zander instruments and machinery,
and capable of being divided by a
twelve servants. The kitchen offices
are in the basement, and an electric
service lift is provided for carrying
food to the upper floors. The hos-
pital is certainly an example of skil-
ful planning on a very confined
space, but it is questionable whether
it would not have been wiser policy
to have found a bigger site so as to
have got better circulation of light
and air about the place.
The building on the east side of
Bolsover Street is, on the ground
floor, entirely occupied by the out-
patient department. The patients
?enter at the north-east angle from a
passage-way leading from Euston
Road, and this also has to serve as
?exit. Close to the entrance is the
porter's lodge and the office, and,
after passing the latter, patients
?enter into a large waiting room. On
one side of this waiting room is a ROYAL MRTlONflL
surgeons' room, each with two ^ ? , ? ,
examining rooms, and at one end QF(THOPffLD/C HOSPITfli
there is a bathroom. The sanitary
?offices for patients lead directly out [30LSOVE.f \ 3? rr
of the waiting hall, one on either side
of the passage leading to the exercise \(l IQ<ZC Q l?ir\MP
room. At the entrance end of the /Yc//l0i_0 llUI IH. /JND
-waiting hall is the operation room, r\i rr P/J"TIF l\l~TC.
with a nurses' or recovery room. /(-/ / I /I I II? \ I
Adjoining, and close to the exit for
patients, are two boot rooms and the -|mii|
?dispensary, also the porter's office. f ? < FIRST FlSOft PLRH
Connected with the mam waiting
hall by a passage is a large exer-
cise and massage room fitted with
Zander instruments and machinery,
and capable of being divided by a
P^1S
ROYAL NATIONAL
GPJHOPfLDIC HOSPITAL
. mLMEJiJA. iv-
\ NUPSLS HOME m
\ GI1T-PATITNTS DEP"-T
\
fi - 47.
GROUND ri^OR PL AN
'January 1, 1910. THE HOSPITAL. 401
folding partition. Off this room are retiring and bath-
rooms, electric and x-ray rooms and surgeons' rooms, with
cloakroom with lavatory. The floors above contain bed-
rooms for 11 sisters and 33 nurses, and six rooms for
servants. In addition to these there are sitting, writing,
and visitors' rooms and home eister'6 room, also a sick ward
for two nurses on the second floor.
The construction of the buildings throughout is of fire-
resisting materials, the floors being made of ferro-concrete
on the Hennebique system. The ward floors are covered
with linoleum stuck down to the concrete; the nurses' and
sisters' rooms are treated in the same way. The floors of
the corridors, sculleries, and sanitary offices are finished
with terrazzo. The operation room has a terrazzo dado,
with opalite tiles on the upper parts of walls and the
ceiling. Fire escape staircases are provided to both build-
ings communicating with all floors.
The architect of both buildings is Mr. Rowland Plumbe>
F.R.I.B.A., of Fitzroy Square, W.

				

## Figures and Tables

**Figure f1:**
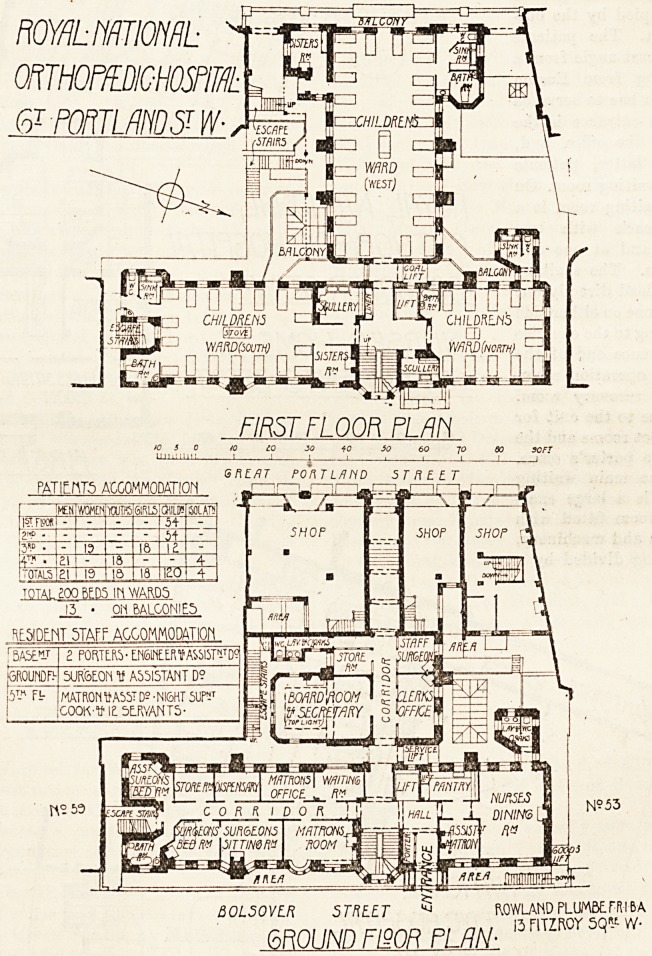


**Figure f2:**